# Effect of COVID-19 precautions on the gut microbiota and nosocomial infections

**DOI:** 10.1080/19490976.2021.1936378

**Published:** 2021-06-16

**Authors:** Armin Rashidi, Maryam Ebadi, Tauseef Ur Rehman, Heba Elhusseini, Harika Nalluri, Thomas Kaiser, Shernan G Holtan, Alexander Khoruts, Daniel J. Weisdorf, Christopher Staley

**Affiliations:** a Division of Hematology, Oncology, and Transplantation, Department of Medicine, University of Minnesota, Minneapolis, MN, USA; b Department of Surgery, University of Minnesota, Minneapolis, MN, USA; c Division of Gastroenterology, Hepatology, and Nutrition, Department of Medicine, University of Minnesota, Minneapolis, MN, USA

**Keywords:** Acute myeloid leukemia, chemotherapy, COVID-19, infection, microbiota

## Abstract

COVID-19 precautions decrease social connectedness. It has been proposed that these measures alter the gut microbiota, with potential clinical consequences. We tested this hypothesis in patients with acute myeloid leukemia (AML) receiving inpatient chemotherapy, a population with extensive exposure to the nosocomial setting and at high risk for infections. Hospitalized patients with AML contributed stool samples to a biorepository protocol that was initiated before COVID-19 and continued without change through the pandemic. Patient-, disease-, and treatment-related characteristics remained the same in the two eras and the only change in clinical care was the implementation of COVID-19 precautions in March 2020. The incidence of all-cause nosocomial infections during the pandemic was lower than in the pre-COVID-19 era. Multivariable analysis revealed an imprint of COVID-19 precautions in the gut microbiota as a viable mechanistic explanation. In conclusion, COVID-19 precautions alter the gut microbiota, thereby mediating pathogen susceptibility and nosocomial infections.

## Introduction

Hospitals around the world have implemented precautionary measures to limit the spread of the severe acute respiratory syndrome coronavirus 2 (SARS-CoV-2), the causative agent of the ongoing coronavirus disease 2019 (COVID-19) pandemic. Precautions include the use of face masks and face shields, social distancing, fewer in-room staff visits, and restricted visitor policies including no food brought from outside the hospital. The effect of these measures on the gut microbiota and its potential clinical consequences are unknown. Understanding gut microbiota changes due to systematic changes in clinical, hygienic, social, or behavioral practice is important because the gut microbiota is a master regulator of host immunity and pathogen susceptibility. Microbial antigens and metabolites communicate with the immune system, eliciting homeostatic microbiota-specific responses that recognize commensal microbes across the gut mucosal barrier. Disruptions in the microbiota-immune system cross-talk can lead to inflammation and aberrant immune responses.^1,2^ Changes in the gut microbiota can increase pathogen susceptibility by at least 3 mechanisms: (*i*) selection for pathobionts, esp. those harboring antibiotic-resistance genes,^3^ (*ii*) altered immune response to microbial antigens (*i.e*., disrupted priming of the immune system by the microbiota),^4^ and (*iii*) decreased production of beneficial microbial products such as short-chain fatty acids with tonic and trophic effects on the gut epithelial barrier.^5,6^

Social distancing may alter the microbiota by decreasing person-to-person microbial transmission.^7,8^ In non-human primates, social interactions are a major determinant of the gut microbiome.^9^ In human societies alike, isolation and socialization in smaller groups (*e.g*., within rather than among families) reduce social contacts, resulting in microbiomes that resemble those of close family members or friends.^10^ The increased use of disinfectants, sanitizers, and antibiotics for containment of the virus has been proposed to cause collateral damage to the gut microbiota, compromising colonization resistance and promoting the growth of antibiotic-resistant species and pathogens.^11^ The “disappearing microbiota hypothesis” predicts that reduced acquisition of microbes due to decreased exposure to the external environment (versatile foods, people, and environment) and increased use of antimicrobial agents will cause microbial diversity loss.^12^ The pandemic has led to changes in eating habits and lifestyle, such as increased consumption of fruits and vegetables and higher adherence to the Mediterranean diet.^13^ Changes to exercise habits resulting from COVID-19 restrictions may also alter the gut microbiota.^14^

Patients with acute myeloid leukemia (AML) receiving chemotherapy represent an ideal population to test this hypothesis. These patients typically spend several weeks in the hospital and suffer a high rate of nosocomial infections. Using high-throughput bacterial sequencing of stool samples collected from patients with AML enrolled in a biorepository protocol that started before COVID-19 and is continuing without change in the COVID-19 era, we investigated the impact of COVID-19 precautions on the gut microbiota and nosocomial infections. Because patient-, disease-, and treatment-related characteristics of the enrolled subjects remained unchanged in the two eras and the only change in standard of care was the implementation of COVID-19 precautions in March 2020, this biorepository uniquely positioned us to identify changes in the gut microbiota and nosocomial infection rates that resulted from COVID-19 precautions.

## Results

Patient age (median [interquartile range, IQR]: 61 [50–72] vs. 59 [53–68] years]), hospitalization length (median [IQR] 32 [25–40] vs. 31 [25–37] days), incidence of neutropenic fever (83% vs. 82%), and use of parenteral nutrition (34% and 36%) were similar in pre-COVID-19 and COVID-19 eras. However, the incidence of microbiologically documented infections before COVID-19 was more than twice higher than during the pandemic (25.9 vs. 11.9 events per 1000 patient-days; Table 1). No particular infection type or organism seemed to drive the observed difference, suggesting that a single underlying regulator of pathogen susceptibility may be involved. The gut microbiota is a master regulator of host immunity and infection susceptibility.^15^ As an example, the expansion of *Akkermansia* in the gut increases the risk of neutropenic fever during anti-leukemia chemotherapy,^16^ an effect that may be mediated by the mucolytic action of *Akkermansia* promoting bacterial translocation to the bloodstream.^17^ Thus, we compared the gut microbiota before and during the pandemic as a potential explanation for different infection rates in the two eras.

**Table 1.** Microbiologically documented infections per 1000 patient-days

Pre-COVID-19 era	COVID-19 era
Total: 25.9 Enterococcal bacteremia: 7.8 Other Gram-positive bacteremia: 5.2 Gram-negative bacteremia: 1.7 *Clostridioides difficile*: 6.9 Invasive fungal infection (Aspergillus): 3.5 Viral infection: 0.8	Total: 11.9 Enterococcal bacteremia: 3.0 Other Gram-positive bacteremia: 3.0 *Clostridioides difficile*: 3.0 Polymicrobial infection: 3.0

**Figure 1.**
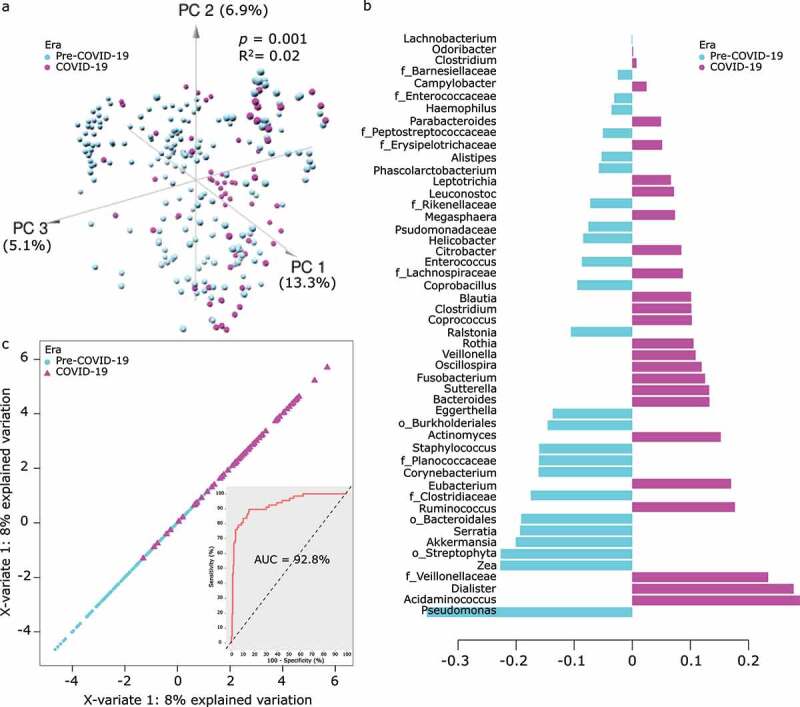
Gut microbiota in pre-COVID-19 vs. COVID-19 era. (a) Principal component analysis using operational taxonomic units and Aitchison’s distance. Each circle represents a stool sample and its color indicates the era in which it was collected. The first 3 principal component (PC) axes are shown, with numbers in parentheses indicating the proportion of total data variation explained by the corresponding axis. *p* value and R^2^ are from an adonis test with 999 permutations. (b) Loadings of the 50 most discriminant taxa on component 1 from sparse partial least squares discriminant analysis (sPLS-DA). Bars to the right indicate differentially abundant taxa in the COVID-19 era and those to the left indicate differentially abundant taxa in the pre-COVID-19 era. The length of each bar indicates the strength of the association. All taxa are at the level of genus, except those with inconclusive genus-level characterization; the latter are shown at the level of family (f) or order (o). (c) Group separation by era using candidate taxa from sPLS-DA listed in panel b. Each item (triangle for COVID-19 era and circle for pre-COVID-19 era) represents a sample. The receiver operating characteristic curve corresponding to the main plot is shown as an inset. AUC: area under the curve

After filtering, we analyzed 263 samples (pre-COVID-19: 196; COVID-19: 67) containing an average of 19,604 high-quality reads per sample. Samples collected in the two eras had a different microbiome composition (PERMANOVA *p* = .001, R^2^ = 0.02; no difference in dispersion, *p* = .78; Figure 1a). To find taxa that made the largest contributions to the discrimination between eras, we performed sparse partial least squares discriminant analysis (sPLS-DA)^18^ on centered log-ratio (clr)-transformed abundances (123 genera) (Figure 1b). Using the 50 most discriminating taxa, an area under the receiver operating characteristic curve of 92.8% for era discrimination was obtained (Figure 1c). The stability of 47 of these taxa in leave-one-out cross-validation was 100%.

**Figure 2.**
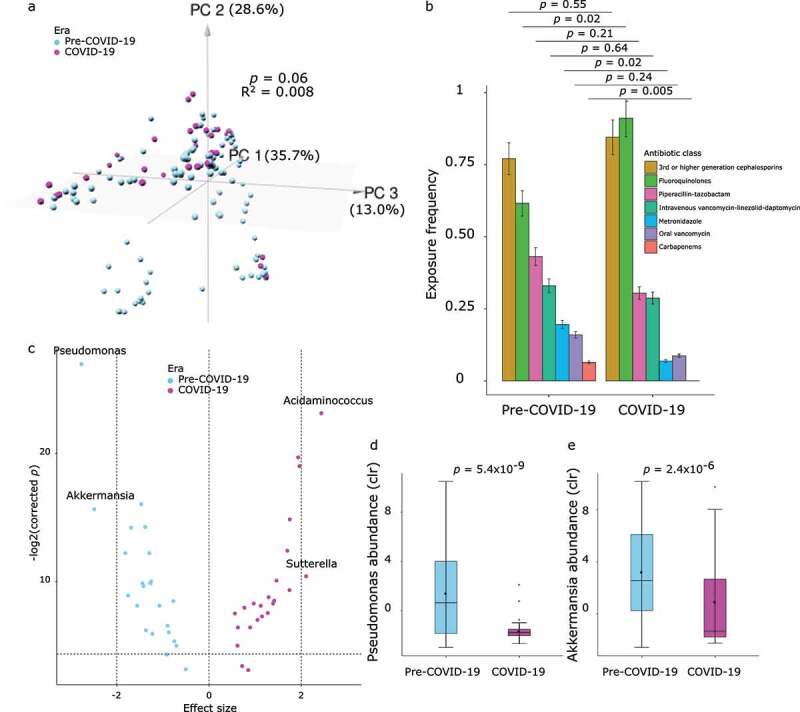
Taxonomic differentiation of pre-COVID-19 and COVID-19 eras in multivariable analysis. (a) Principal component analysis applied to the antibacterial antibiotic exposure history of the samples. Each circle represents a stool sample and its color indicates the era in which it was collected. The first 2 axes are shown, with numbers in parentheses indicating the proportion of total data variation explained by the corresponding axis. *p* value and R^2^ are from an adonis test with 999 permutations. Samples with an identical antibiotic history are superimposed, visually creating fewer data points than the actual number of samples. (b) Antibacterial antibiotic exposures in the two groups. Seven common classes of antibiotics were considered. *p* values are from chi-squared tests with Fisher’s exact test when appropriate. (c) Volcano plot showing association between taxa abundances and era in multivariable linear regression. clr-transformed taxa abundances were the dependent variables in separate models, with era (COVID-19 vs. pre-COVID-19) as a binary predictor and sample collection day and the first two axes of antibiotic history as covariates. Samples were the units of analysis. Each circle represents a taxon. Only the 100% stable taxa from sPLS-DA were considered. The regression coefficient for era was considered its effect size and plotted along the x-axis. A positive (negative) effect size means that the corresponding taxon is associated with COVID-19 (pre-COVID-19) era. The *p* value corresponding to the regression coefficient for era was corrected for multiple testing and plotted along the y-axis after logarithmic transformation. The horizontal dashed line indicates a corrected *p* value threshold of 0.05. The points above this line indicate statistically significant taxa. The two vertical dashed lines on the sides indicate thresholds of −2 and 2 for the effect size to define a strong association. Points to the right of x = 2 or to the left of x = −2 represent taxa that are strongly associated with era. Taxon identity is shown for strong significant taxa. (d-e) Univariate comparison between the two eras for strong significant taxa in panel c which were also significant in all leave-one-patient-out runs. *p* values are from t-tests

Next, we used the clr-transformed abundances of the 47 candidate taxa in sPLS-DA as dependent variables in separate multivariable linear regression models with era being a binary predictor. We quantified the “antibiotic history” of each sample by considering the time series of exposures to 7 major classes of antibacterial antibiotics between hospital admission and the day the sample was collected. For a given day, if a given antibiotic was used, exposure to that antibiotic was coded 1 and otherwise, zero. Next, we applied a decaying average function to the antibiotic history of each sample – a time series of 0’s and 1’s for each antibiotic class – to achieve a single numerical value summarizing the exposure history of the given sample for the given antibiotic. The decaying average method flexibly models both recent and less recent exposures by placing more weight on exposures in the more recent days preceding the sample. In principal component analysis (PCA) using antibiotic histories, a slight separation of samples belonging to the two eras was apparent (PERMANOVA *p* = .06, R^2^ = 0.008; Figure 2a). This separation was primarily driven by higher exposure to fluoroquinolones (*p* = .02, t-test on decaying averages) in the COVID-19 era and lower exposure to metronidazole (*p* = .02), and carbapenems (*p* = .005) in the pre-COVID-19 era (Figure 2b). Therefore, we included the first two PCA axes of antibiotic history along with sample collection day relative to day 1 of chemotherapy as covariates in the regression model. In this analysis, era was a strong (absolute value of regression coefficient >2) and significant (corrected *p* < .05) predictor of four taxa. Two taxa (*Pseudomonas* and *Akkermansia*) were associated with the pre-COVID-19 era and two (*Acidaminococcus* and *Sutterella*) were associated with the COVID-19 era (Figure 2c). Decreasing the decay rate from 2 to 1.5 to increase the relative importance of antibiotic exposures in less recent days did not change the results (Supplementary Fig. 1). Finally, because most patients contributed more than one sample, we explored the possibility of our results being driven by samples from a single patient. To evaluate this possibility, we re-ran the regression models on subsets of the database, each lacking samples from a different patient. Reassuringly, two of the four genera from the previous step (*Pseudomonas* and *Akkermansia*) remained the only two taxa with an effect size larger in absolute value than 2 and corrected *p* < .05 in 100% of the runs (univariate comparisons shown in Figure 2d,e).

**Figure 3.**
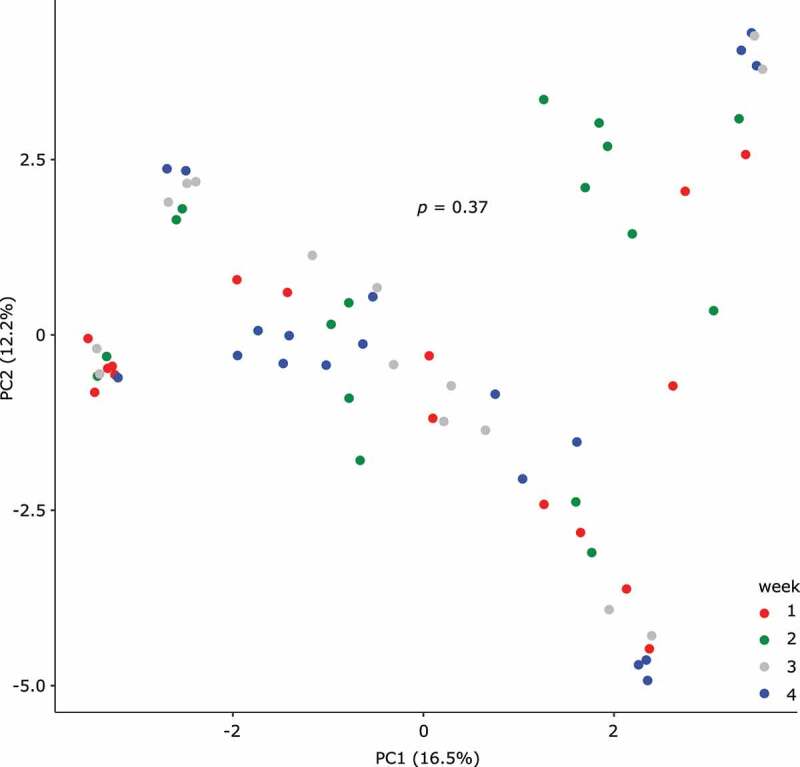
Microbiota composition over time in the COVID-19 era. Samples collected in the COVID-19 era were classified in the PCA space (top two principal components) according to the week they were collected relative to day 1 of chemotherapy. Each circle represents a stool sample and its color indicates the week of collection. The numbers in parentheses indicate the proportion of total variation explained by the corresponding axis. The *p* value is from a *betadisper* test with 999 permutations

Because COVID-19 precautions were implemented at our hospital in one step, the effect of individual components of these precautions on the gut microbiota cannot be determined. Considering the well-established effects of diet on the gut microbiota,^19,20^ we explored the possibility that the “no food from outside” policy might have been the primary determinant of microbiota differences between the two eras. First, an informal survey of 3 nursing staff who took care of our acute leukemia patients in both eras suggested that even in the pre-COVID-19 era, more than 80% of meals were ordered from the hospital food services. Next, we reasoned that the new food policy could reduce gut microbiota variability caused by different food preparations in different outside sources, thus the microbiota of different samples would become more similar over time in the COVID-19 era. We tested this hypothesis by comparing the compositional dispersion of samples collected in different weeks of chemotherapy (week 1: hospitalization through day 7 of chemotherapy; week 2: days 8–14, week 3: days 15–21, week 4: day 22 and later). This analysis, done using *betadisper* in R, did not support a change in dispersion over time (Figure 3; *p* = .37, 999 permutations).

## Discussion

Our study represents a before/after comparison where the only difference in clinical care between the two eras was the implementation of COVID-19 precautions during the pandemic. The incidence of nosocomial infections was lower during the pandemic, a finding potentially explained by gut microbiota changes. We found a lower abundance of *Pseudomonas* and *Akkermansia* in the gut microbiome during the pandemic. By thinning the mucus layer, *Akkermansia* potentiates pathogen translocation to the bloodstream.^16,17^ Similarly, the PA-I lectin of *Pseudomonas aeruginosa* impairs tight junction integrity of the intestinal barrier, promoting gut-derived sepsis in stressed hospitalized patients.^21^ Considering that the standards of leukemia care did not change over the lifetime of this study, COVID-19 precautions are the most likely cause of the observed changes in the microbiota. Importantly, we adjusted our analysis for antibiotic exposures, a known cause of microbiota changes. The extent to which the gut microbiota mediated the effect of COVID-19 precautions on nosocomial infection rates is not clear because a direct effect of the implemented measures on reducing clinical infections is also possible.

Decreased person-to-person and environment-to-person transmission of specific taxa can result in their lower abundance in the gut microbiota. *Pseudomonas* colonizes medical devices and water and can be transmitted from the environment or other individuals to the patient.^22^ Although the ability of ingested *Pseudomonas* to reach and colonize the colon is unknown, decreased transmission resulting from COVID-19 precautions is a potential explanation for our findings. Alternatively, reduced microbial transmission could make the microbiota less accommodating to specific taxa. *Akkermansia* resides in the mucus layer and uses mucin as its sole carbon and nitrogen source.^23^ Short-chain fatty acids, produced by several commensal members of the gut microbiota, stimulate mucin production by goblet cells.^24^ As a possible mechanism, a reduction in such bacteria resulting from decreased transmission could make the gut a suboptimal environment for *Akkermansia*. Direct transmissibility of *Akkermansia* has not been evaluated.

Thus far, the effect of COVID-19 precautions on the gut microbiota has been an attractive, but largely untested, question. Our study supports this notion in an extreme nosocomial scenario where heavily immunosuppressed patients are exposed to the healthcare personnel and environment for a prolonged period. The extent to which these findings can be generalized to less ill patients with less intense nosocomial exposure requires further research. Another unclear aspect of our findings is the durability of the observed changes in the gut microbiota and their clinical consequences in the outpatient setting and for the next phases of treatment. Finally, and considering microbiome transmissibility among close contacts,^10^ nosocomial changes in the gut microbiota may impact the microbiome of the patients’ families and socializing partners after discharge from the hospital, with potential consequences for the “social” and “communal” microbiome.^8^ We postulate that society-level COVID-19-related precautions may prevent rapid dilution and ultimate resolution of nosocomial changes in the gut microbiota.

In a previous study of patients with acute leukemia, we showed that the expansion of *Akkermansia* within the gut microbiota predicts higher levels of flagellin in the serum in subsequent days.^16^ This observation suggested that the mucus-thinning effect of *Akkermansia* may promote translocation of motile bacteria to the bloodstream. In this context, our finding of decreased *Akkermansia* in the COVID-19 era may indicate a reduced risk of bloodstream infection during chemotherapy. In contrast, *Akkermansia* has a plethora of long-term beneficial impacts on the host such as anti-inflammatory effects and protective effects against metabolic syndrome.^25,26^ Therefore, if the reduction in *Akkermansia* in our patients is durable, it may have long-term detrimental effects on the host.

In conclusion, COVID-19 precautions changed the gut microbiota and led to a lower incidence of nosocomial infections in patients with AML. This study supports the theory that social distancing influences the gut microbiota, with potential clinical consequences. In addition, our findings indicate that microbiome results obtained during the pandemic may not be directly comparable to earlier results.

## Patients and methods

### Biorepository

Adult patients with newly diagnosed or relapsed/refractory AML receiving inpatient chemotherapy were eligible for this biorepository protocol (ClinicalTrials.gov NCT03316456). The protocol was approved by the University of Minnesota Institutional Review Board. Written informed consent was obtained from participants prior to inclusion in the study. An expected ~4 weeks of hospitalization was required. No other inclusion or exclusion criteria were used. Stool samples were collected twice weekly (Mondays and Thursdays ±1 day window) between hospital admission and day 28 of chemotherapy or discharge (whichever occurred first). Supportive care including antibiotic stewardship and nutrition remained unchanged throughout the study, with the exception of the following COVID-19 precautions that were initiated on March 20, 2020: (*i*) universal face masking in the hospital, (*ii*) mandatory use of face mask and face shield by the treatment team, (*iii*) “no visitor” policy, and (*iv*) “no food from outside” policy. Patients continued to order food from the hospital’s food services, which maintained an unchanged menu. However, more orders included individually packed or boxed items. Our antibiotic stewardship recommends acyclovir for viral, an azole for fungal, and levofloxacin for bacterial prophylaxis for the duration of neutropenia. Bacterial prophylaxis is continued until the development of neutropenic fever or first neutrophil count rise above 1x10^9^/L, whichever occurs first. When oral intake decreases to <60% of the lower limit of estimated energy and protein needs for 7 days, we generally initiate parenteral nutrition. Patients are asked to avoid flossing and to use soft toothbrushes. Stool samples were collected in 95% ethanol-filled sterile tubes and stored at −80°C.

### 16S ribosomal RNA (rRNA) gene sequencing

DNA was extracted using the DNeasy PowerSoil DNA isolation kit (QIAGEN, Hilden, Germany). The V4 hypervariable region of the 16S rRNA gene was amplified on an Illumina MiSeq platform (2 x 300 paired-end mode) by the University of Minnesota Genomics Center. Sequences were processed in QIIME 2.^27^ Quality filtering, adaptor trimming, and stitching of raw sequences were done using the quality control pipeline SHI7 (trim threshold 32, threshold of Q37).^28^ Paired ends were merged using FLASH.^29^ Operational taxonomic unit (OTU) picking was done using NINJA-OPS (default parameters and 97% similarity threshold) and the Greengenes database; Bowtie2 was used for alignment.^30–32^ OTUs with a frequency <0.01% of the reads and samples with fewer than 500 reads were removed. The BIOM table was exported from QIIME into R 3.4 (Vienna, Austria). Raw sequence reads were uploaded to the NCBI Sequence Read Archive and are accessible under BioProject ID SRP141394. Sample identifiers in the online database appear as “7D” (title of our acute leukemia unit) followed by patient number followed by collection date. For example, 7D069_28Nov20 indicates a sample collected on Nov 28, 2020 from patient number 69. Further details can be obtained from the corresponding author upon request by e-mail.

### Statistics

All analyses were performed using custom scripts and *phyloseq, vegan*, and *mixOmics* packages in R. Samples, rather than patients, were the units of analysis throughout. Ordination using Aitchison’s distance and centered log-ratio (clr) transformed taxa abundances^33^ was visualized by principal component analysis (PCA) as an unsupervised method. clr transformation is suitable for the analysis of compositional data.^33^ We compared microbiome composition between samples from the two eras by permutational multivariate analysis of variance (PERMANOVA)^34^ with an adonis test and 999 permutations. We compared microbiome dispersion among samples collected in different weeks of chemotherapy using *betadisper* in R. To identify the most discriminatory taxa between the two eras, we performed sparse partial least squares discriminant analysis (sPLS-DA) as a supervised method. To avoid overfitting, we decided *a priori* to choose the 50 most discriminant genera in component 1 for further analysis. Loading weights were determined using maximal mean values of the contribution by the corresponding taxa. The performance of the model was assessed by leave-one-out cross-validation and the stability of the selected genera across cross-validation folds was determined. The list of the most discriminant genera with 100% stability was stored for multivariable regression (see below).

We quantified the “antibiotic history” of each sample using the time series of exposures to 7 major classes of antibacterial antibiotics between hospital admission and the day the sample was collected. The antibiotic classes considered were fluoroquinolones, third or higher generation cephalosporins, metronidazole, piperacillin-tazobactam, intravenous vancomycin/daptomycin/linezolid, oral vancomycin, and carbapenems. For a given day, if a given antibiotic was used, it was coded 1 and otherwise, zero. Day 0 was defined as the first day of chemotherapy. Next, we applied a decaying average function to the time series of 0’s and 1’s for each antibiotic class to achieve a single numerical value summarizing the exposure history for the given sample and antibiotic. As an example, if levofloxacin was used on days 1–3 for a patient admitted on day −1 (one day before starting chemotherapy), the time series for levofloxacin for a sample collected on day 5 of chemotherapy from this patient would be (0,0,1,1,1,0), indicating that the antibiotic was not used on days −1, 0 (first day of chemotherapy), and 4, but was used on days 1, 2, and 3. With a decay factor of 2, the levofloxacin history for this sample would be quantified and summarized as 0 × 2° + 1×2^−1^ + 1 × 2^−2^ + 1 × 2^−3^ + 0 × 2^−4^ + 0 × 2^−5^ + 0 × 2^−6^ = 0.875. With this decay factor, exposure on a given day receives twice higher weight than exposure on the previous day. A smaller decay factor would make the weights assigned to antibiotic exposures along time more homogenous. The first two axes of PCA applied to antibiotic histories were stored for multivariable regression (see below). We tested two values of the decaying factor, namely 2 and 1.5, as a sensitivity analysis.

Finally, we built multivariable linear regression models to predict the abundance of the most discriminant taxa from sPLS-DA by era. A separate model was built for each selected taxon (clr-transformed abundance) as the dependent variable, using era as the binary predictor. The first two PCA axes of antibiotic history and sample collection day relative to day 1 of chemotherapy were included as covariates. To generate volcano plots, the regression coefficient for era was considered the effect size and plotted along the x-axis, while its corresponding logarithmically transformed *p* value, corrected by the Benjamini-Hochberg method^35^ due to multiple taxa/models, was plotted along the y-axis. A threshold of 0.05 for the corrected *p* value was used to define statistical significance and a threshold of 2 for the absolute value of the effect size was used to define a strong association. In the last step, we explored the possibility of our results being driven by samples from a single patient by re-running the regression models on subsets of the database, each lacking samples from a different patient. A taxon was considered significant if in all the runs its corresponding corrected *p* value and absolute effect size were <0.05 and >2, respectively.

## Supplementary Material

Supplemental Material

## Data Availability

Raw sequence reads were uploaded to the NCBI Sequence Read Archive and are accessible under BioProject ID SRP141394 (https://www.ncbi.nlm.nih.gov/sra/?term=SRP141394).
