# Supplementary figures and images for: Effect of COVID-19 precautions on the gut microbiota and nosocomial infections
Source: Gut Microbes. 2021 Jun 16;13(1):1936378. doi: 10.1080/19490976.2021.1936378 (PMC8210870; doi:10.1080/19490976.2021.1936378)

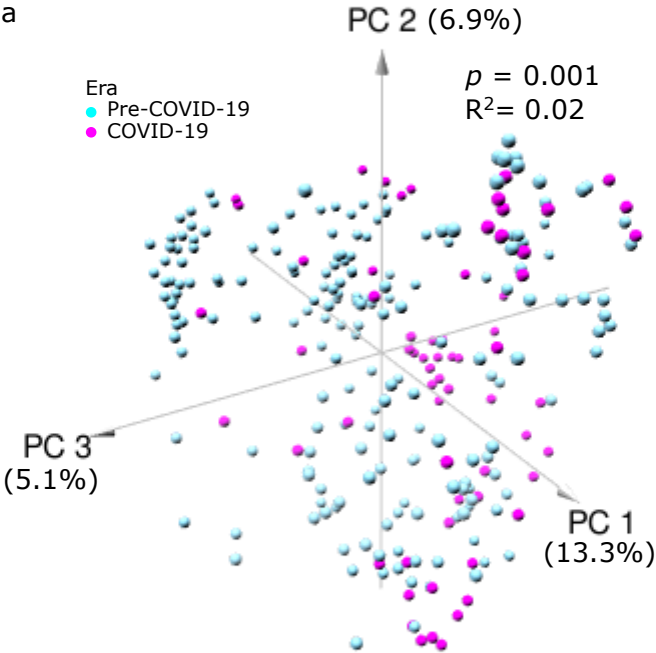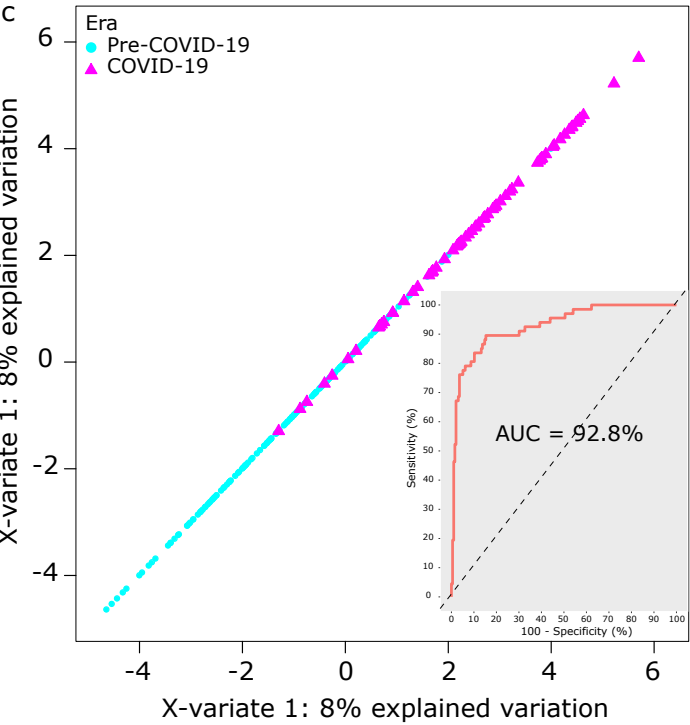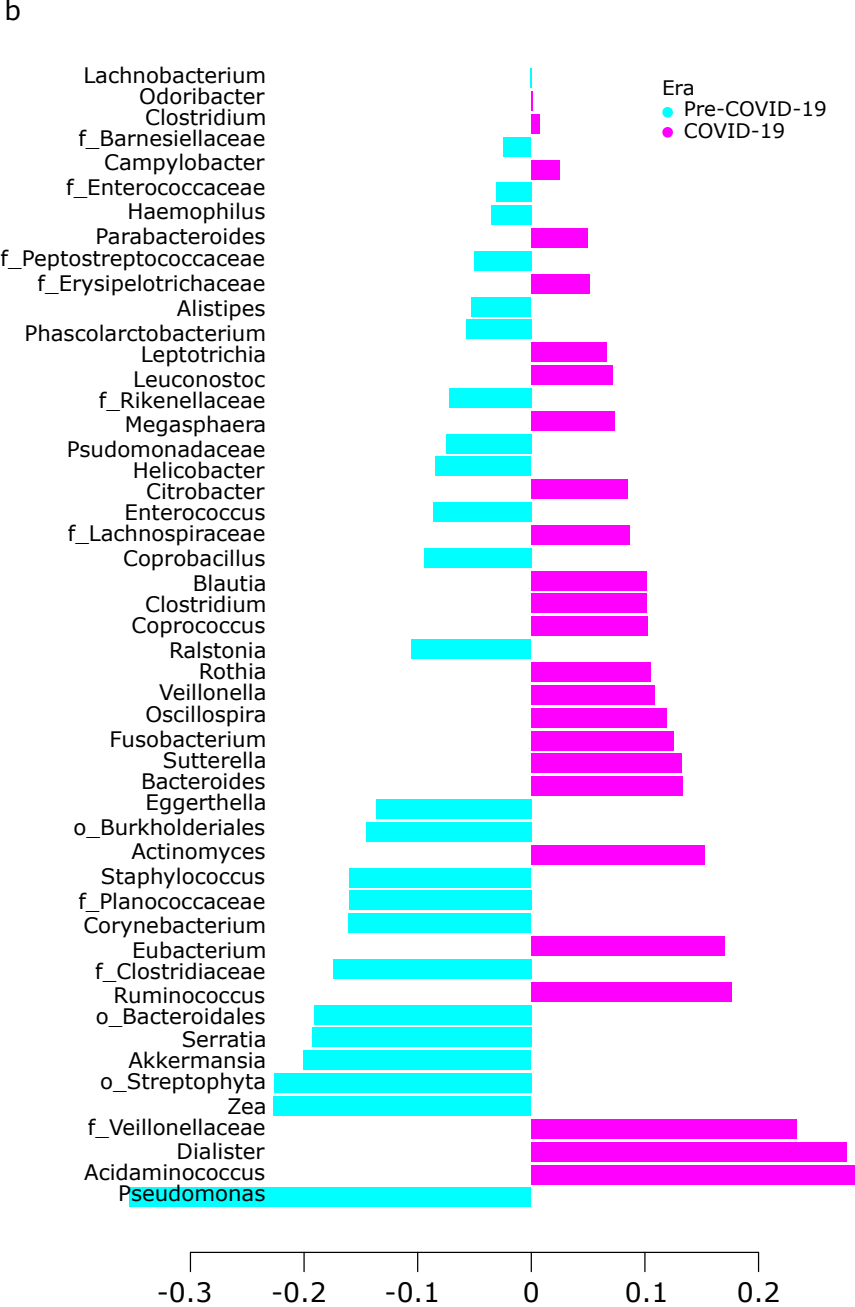

Supplement: Supplemental Material [file KGMI_A_1936378_SM5427.zip › supplementary/downloadFromZipFile.pdf]
